# "Shelters" and Sanitation

**Published:** 1897-03-06

**Authors:** 


### "SHELTERS" AND SANITATION.

On February 10th a paper was read at the Sanitary Institute by Dr. Waldo on "The Sanitary Supervision of Shelters for the Homeless." He said that the alternatives open to the penniless were sleeping out in the open, the vagrant ward, and the free night shelter. Practically, however, it was not the absolutely destitute who were catered for by the shelters; it was the patronage of the "pennied" poor they aimed at, and in seeking this they came into sharp trade competition with the common lodging-housekeepers, in which they gained a huge advantage from the fact that they were under no obligation to conform to the regulations which were enforced in regard to common lodging-houses. These must be kept in good maintenance and repair, and were under strict supervision, whereas the shelters were a kind of no man's land, and the medical officer of health could only deal with them as he did with private property in general. Shelters were great centres for the dissemination of small-pox, itch, and other infectious diseases, and for the spread of body vermin. After the reading of the paper the following resolution was passed: "That, in the opinion of this meeting, all night shelters for the homeless should be placed within the provisions of the Common Lodging Houses Act."

